# Investigating CTL Mediated Killing with a 3D Cellular Automaton

**DOI:** 10.1371/journal.pcbi.1000466

**Published:** 2009-08-21

**Authors:** Frederik Graw, Roland R. Regoes

**Affiliations:** Institute of Integrative Biology, ETH Zurich, Zurich, Switzerland; Utrecht University, Netherlands

## Abstract

Cytotoxic T lymphocytes (CTLs) are important immune effectors against intra-cellular pathogens. These cells search for infected cells and kill them. Recently developed experimental methods in combination with mathematical models allow for the quantification of the efficacy of CTL killing *in vivo* and, hence, for the estimation of parameters that characterize the effect of CTL killing on the target cell populations. It is not known how these population-level parameters relate to single-cell properties. To address this question, we developed a three-dimensional cellular automaton model of the region of the spleen where CTL killing takes place. The cellular automaton model describes the movement of different cell populations and their interactions. Cell movement patterns in our cellular automaton model agree with observations from two-photon microscopy. We find that, despite the strong spatial nature of the kinetics in our cellular automaton model, the killing of target cells by CTLs can be described by a term which is linear in the target cell frequency and saturates with respect to the CTL levels. Further, we find that the parameters describing CTL killing on the population level are most strongly impacted by the time a CTL needs to kill a target cell. This suggests that the killing of target cells, rather than their localization, is the limiting step in CTL killing dynamics given reasonable frequencies of CTL. Our analysis identifies additional experimental directions which are of particular importance to interpret estimates of killing rates and could advance our quantitative understanding of CTL killing.

## Author Summary

The immune response mediated by cytotoxic T lymphocytes (CTLs), which kill infected cells, is thought to be essential to control viral infections. Experiments offer data which allow one to address the efficacy of this cell population *in vivo* and to estimate characterizing parameters. However, it is unclear which mathematical description reflects the experimental situation best and leads to reliable parameter estimates that quantify CTL efficacy. We simulate the spatial interaction of CTLs and infected cells in a 3-dimensional computer model to examine different mathematical descriptions of the experimental situation, independently of experimental data. Thereby we find an appropriate mathematical term to describe the killing process. Estimates obtained so far describe CTL efficacy on a population level. By varying the individual properties of simulated CTLs, such as the velocity, we find that the time a CTL needs to kill an infected cell is probably the key factor limiting CTL killing efficacy. Our analysis identifies additional experimental directions which could advance our quantitative understanding of CTL killing for different diseases.

## Introduction

Cytotoxic T lymphocytes (CTL) are some of the most important cells of our immune system. They are particularly important against viral infections or tumours. They recognize infected cells by scanning their surfaces for peptide-MHC-I complexes which present peptide fragments sampled from the cytoplasm. These complexes can tell the CTL if the cell is infected or not. Once activated and primed for a specific peptide-MHC-I complex, CD8^+^ T cells differentiate into effector CTL, which are able to lyse infected cells. After an infection is cleared, some specific CTL may persist as memory cells.

Immunologists are interested in quantifying the efficacy of CTL *in vivo*. An appropriate measure of CTL efficacy would allow us to disentangle quantitative from qualitative aspects of the CTL response: For example, such a measure should tell us whether a memory CTL response is less efficacious than an effector CTL response because there are fewer cells, or because individual memory CTL do not perform as well as effector cells. A measure of CTL efficacy represents the first step in predicting if CTL responses will be able to control an infection, and in quantifying the selection pressure CTL responses exert on the pathogen population. This selection pressure may lead to immune escape where the virus evolves to become mainly undetected by the actual immune response [1],[2]. Rates which determine how fast CTL lyse infected cells are already estimated for HIV-I *in vitro* [3] and indirectly via the selective advantage of escape variants *in vivo* [4].

The best experimental data for the estimation of the CTL efficacy *in vivo* so far originate from the *in vivo* CTL killing assay [5],[6]. In this assay, cells are prepared to display LCMV-peptides on their MHC-I molecules. The cells are then transferred into mice which harbour CTL specific for these LCMV-peptides. It is known that the transferred cells migrate to the spleen where they are targeted by CTL. These cells are mostly located either in the *red pulp* or in the T cell-zones (*perioarteriolar lymphoid sheaths (PALS)*) depending on the stage of infection [7]. While effector CTL preferentially accumulate in the *red pulp*, memory CTL are mostly located in the *PALS*. Some time after the transfer, the levels of target cells are determined in the spleen. To estimate CTL efficacy, Regoes et al. [8] and Yates et al. [9] proposed a mathematical model that takes into account the migration of target cells into the spleen, and their subsequent killing by CTL. Fitting this model to *in vivo* CTL killing data, we obtained a killing rate constant 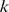, and proposed this constant as a measure for CTL efficacy. We found differences between killing rate constants of effector and memory CTL, as well as for immunodominant and -subdominant epitopes (see Table 2 in [9]).

In these previous studies, we intended to compare the efficacy of distinct CTL populations whose levels differ. Therefore we assumed a mass-action killing term to disentangle quantitative from qualitative aspects of CTL killing. However, the validity of the mass-action assumption is uncertain. Furthermore, it is unclear how the killing rate constant in our mathematical model, which describes CTL killing on the level of the cell populations involved, is related to properties of individual CTL. For example, how does CTL velocity or the time needed to kill a cell influence the estimate of the killing rate constant?

To address these questions we simulate the dynamic inside the spleen or *PALS*, respectively with a three-dimensional cellular automaton (CA) [10],[11]. A CA is an individual-based computer simulation of a dynamical system on a lattice (in our case a three dimensional one). This method allows us to identify a more appropriate mathematical description of CTL killing than the simple mass-action term. Additionally, by generating *in vivo* CTL killing data for different scenarios, we are able to relate the properties of individual cells to the population dynamics of the system.

We find that there is a parameter regime in which the behaviour of our CA model of CTL killing in the spleen is consistent with data obtained by two-photon microscopy [12]–[15]. Further, we find that the most appropriate mathematical description of CTL killing is linear in the target cell levels, and a saturating function of the CTL levels. However, fitting a mathematical model with such a saturating killing term does not improve the fit to the original *in vivo* CTL killing data consistently.

Studying the influence of single cell properties on our killing rate estimates we find that one specific experimental detail, which concerns the fate of CTL-target cell conjugates after splenectomy, is of particular importance to be able to interpret the population-level killing rate constants in terms of single cell efficacy. Nevertheless, given the CTL frequencies observed experimentally, the killing rate constant is mainly determined by the time a CTL needs to kill its target, and not the CTL's velocity.

## Results

### A spatial model for the T cell-zone of the spleen

The spleen is the secondary lymphoid organ which surveys the blood for foreign antigen. It consists of *red pulp*, which is a site of red blood cell destruction and comprises roughly 80% of the splenic volume, interspersed with lymphoid regions (*white pulp*). While most of the blood will bypass the lymphoid regions and remain in the direct circulation, around ten percent of the cells will diffuse through the T cell-zone (*PALS*) [16]. During this passage the cells are under constant surveillance by T lymphocytes.

**Figure 1:**
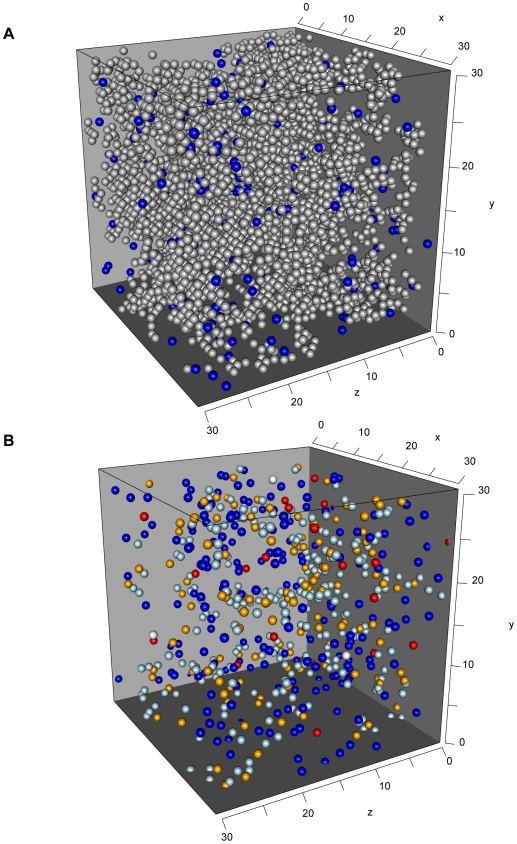
Snapshots of the simulated 3D cellular automaton. (A) CA at the beginning showing CTL (blue) and reticular network (grey). Other cell types are not shown. (B) CA in the middle of a simulation showing target cells (red) and CTL (blue). Target cells in contact to a CTL are labeled in orange, bound CTL in lightblue.

We simulate the population dynamics of the cells in the *PALS* as a cellular automaton. A cellular automaton allows us to investigate the impact of individual cell properties and spatial aspects on the dynamics. Into our simulation model, we incorporate target cells, target-cell-specific CTL, splenocytes, and a limited number of large cells which correspond to dendritic cells or macrophages. In addition, we include the reticular network (RN), which defines the anatomical structure of the spleen, as well as some free space (see Fig. 1). For a detailed description of the automaton see *Materials and Methods*.

**Figure 2:**
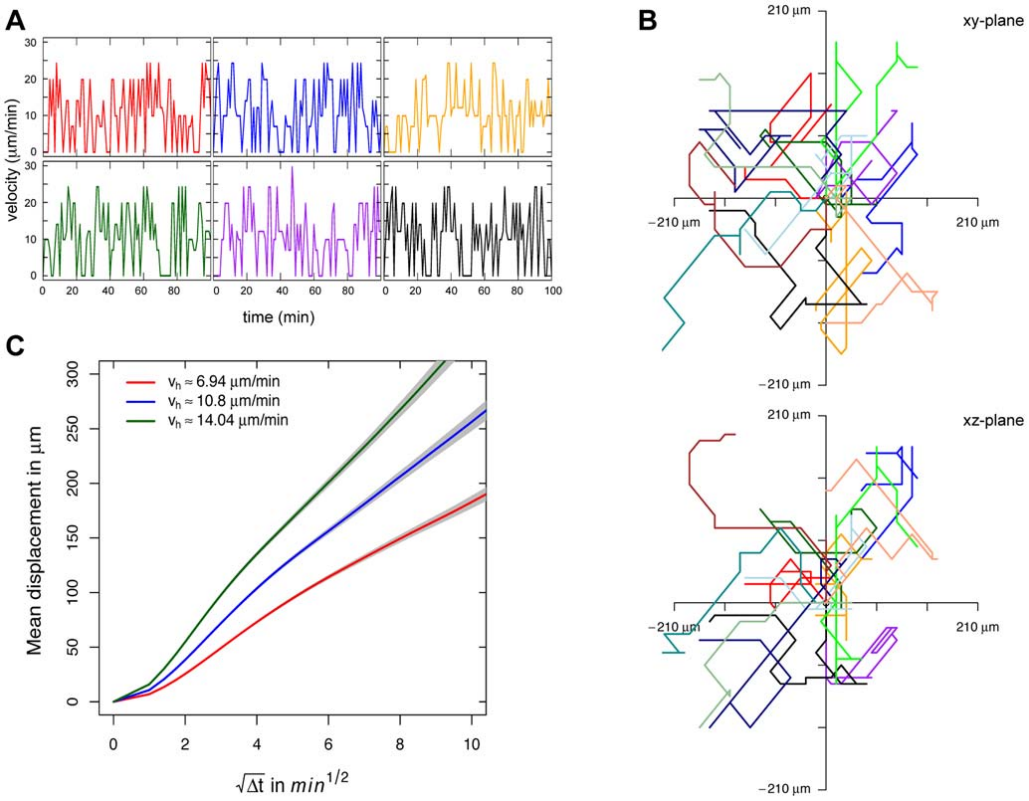
Velocity and motility characteristics of simulated CTL. (A) Velocity fluctuations of six simulated CTL given an average velocity of 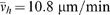 over 100 minutes in the absence of killing. (B) Projection of the tracks of 12 CTL of the same parametrization chosen at random onto the xy- and the xz-planes aligned such that all cells start at the origin. Tracks are plotted for a time period of 60 min. (C) Mean displacement against square root of time for different values of 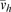 (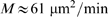 (red), 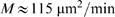 (blue), 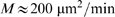 (green)). The blue line corresponds to the parametrization used in (A) and (B).

We first simulate the specific CTL without target cell interaction to characterize their behaviour with regard to experimental observations. The simulated CTL perform a random walk (see Fig. 2B, Video S1 in the *Supporting Material*) consistent with observations made in lymph nodes and the spleen based on *in vivo* imaging techniques [17]–[21].

We are able to manipulate the motility of our simulated CTL through the rules of movement. We adjusted these rules of movement such that they display a mean velocity, velocity fluctuations, and a motility coefficient largly consistent with observations *in vivo*.

Miller et al. [14],[21] measure an average velocity for T cells of about 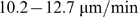 in lymph nodes. It is thought that the velocity is in the range of 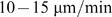 [22]. For the spleen, T cell velocities are observed which are slightly slower, even correcting for differences in the observation method [20], but comparable to those found in lymph nodes [19]. We mainly use a parametrization where the simulated CTL migrate with an average velocity of 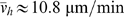.

In Fig. 2A we show the velocity fluctuations of six simulated CTL for this parametrization chosen at random for a time period of 100 min. The amplitude of the velocity fluctuation of the simulated CTL agrees with experimental observations (e.g. [12]). However, the velocity fluctuations are less rugged than those observed in experiments (compare to Fig. 5 in [12]). This is due to the discreteness of space in our cellular automaton, i.e. cells can not be arbitrarily displaced but have to occupy a node in the lattice.

As stated earlier, the simulated CTL perform a random walk. This can be seen from a projection of their normalized tracks (Fig. 2B) as well as from the relation between their mean displacement and the square root of time (Fig. 2C). The discreteness of space also affects the random walk characteristics of simulated CTL. As CTL have to “move” on given edges, there is only a discrete number of turning angles 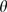 available. As cell movement involves the restructuring of the actin-filament network in the cytoskeleton [23], cells will prefer small turning angles. Therefore, in the simulation, they are programmed to preferentially choose 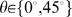 per move. In the absence of killing and given a mean velocity of 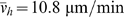, the simulated CTL show a mean turning angle of 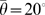 where the turning angle was measured every minute. This slightly increases if we include killing activity (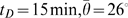). The distribution of 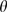 (see Fig. S1) differs from those observed experimentally [13]. This is provoked by the fact that the motility of simulated CTL is only affected by environmental conditions. CTL do not change their moving direction 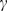 as frequently which leads to a low mean turning angle.

A second variable to characterize cell movement is the motility coefficient, 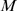. Given standard parametrization, the motility coefficient of simulated CTL is approximately 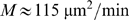 (see Fig. 2C, *blue line*), which is slightly above the range of 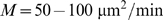 observed experimentally for T cell movement [12]–[15],[22]. For other parametrizations, which we consider in this paper, the motility coefficient is in the range 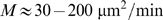.

**Figure 3:**
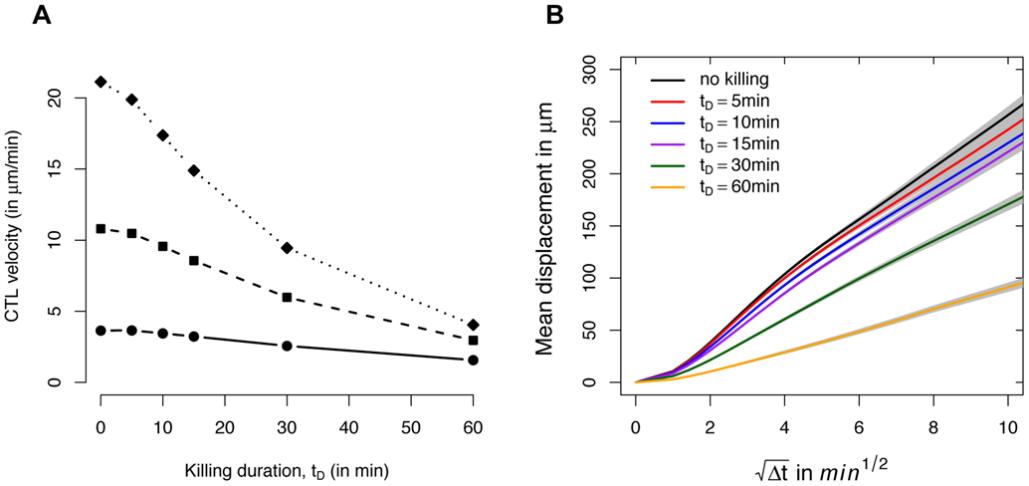
Changes in CTL velocity and motility due to killing activity. (A) Change in mean CTL velocity due to killing activity with different values of 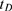 for three different starting velocities 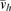 (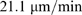 (diamond), 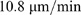 (square), 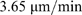 (circle)). (B) Change in mean displacement of simulated CTL with killing activity against the square root of time for different values of 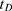 (basic value with no killing (black), 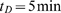 (red), 10 min (blue), 15 min (purple), 30 min (green), 60 min (orange)). The CTL hunting velocity is fixed with 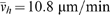.

The mean velocity as well as the motility of CTL decreases in the presence of CTL-target cell interaction (Fig. 3) which is also observed experimentally in the spleen [19]. For CTL-target cell interaction, target cells will appear in the cellular automaton with a certain rate and are killed after encountered by CTL (see *Material and Methods* for details). In our simulations, the mean CTL velocity decreases exponentially in the killing duration 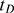 with a rate constant of approximately −0.012 min^−1^, given a fixed CTL concentration. In Fig. 3B we show the change in the mean displacement per square root of time for different values of 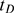 in comparison to the mean displacement of simulated CTL in the absence of killing. In all cases, CTL velocity was fixed to 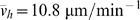. Given a killing duration of 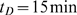, the motility coefficient 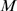 decreases from 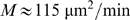 to 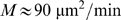. The motility coefficient includes only 15% of the value measured in the absence of killing, if we assume a killing duration of 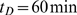. The decrease of 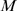 is linear in 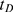.

### Is the mass-action killing term appropriate?

In previous studies [8],[9], it is assumed that the rate at which target cells are killed depends linearly on the frequency of the CTL, 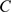, and the frequency of the targets, 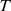, in the spleen. Such a dependence is commonly referred to as mass-action hypothesis. However, the mass-action assumption may be inaccurate if the system is not well-mixed and the dynamics is spatially confined. In addition, the fact that CTL cannot seek for target cells while bound in a conjugate may lead to deviations from a mass-action killing term.

**Figure 4:**
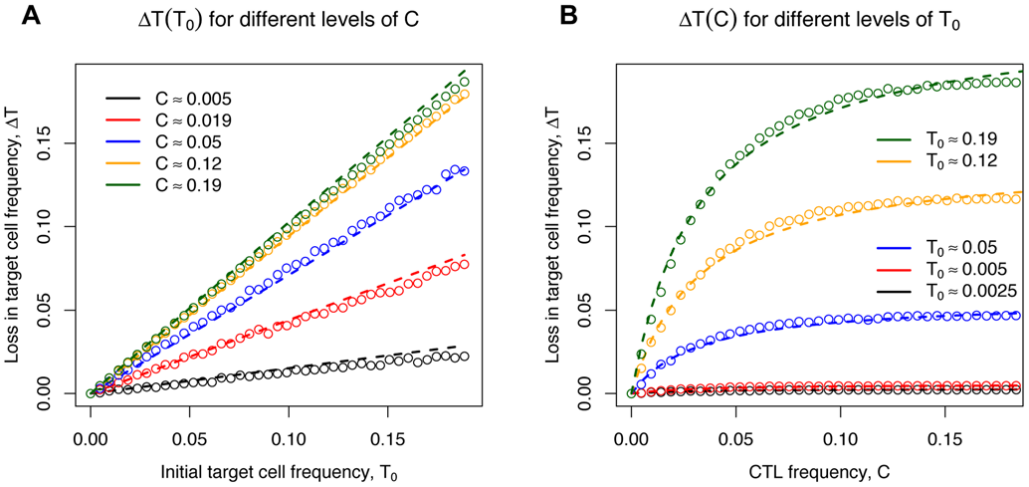
Change in the loss of target cell frequency, 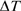. The change in the loss of target cell frequency for different initial levels of target cells, 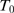, or CTL, 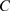. 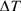 is linear in 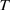 (shown in A) and not linear in 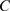 (shown in B). Added lines represent the fit of 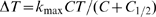 (dashed line).

To address the question whether the mass-action hypothesis appropriately describes the killing dynamics given spatial confinements, we initialized the cellular automaton with different combinations of 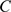 and the starting target cell frequency 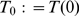. The CTL frequency 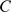 ranges from 0–20% of simulated cells, which covers the frequencies observed for dominant and subdominant effector and memory responses (Tab. 1). The average velocity of CTL, 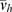, was fixed and the killing duration was defined by 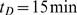, in agreement to experimental observations [24]. The loss of target cell frequency, 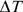, was calculated by
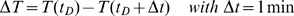
(1)
Fig. 4A shows linearity of 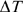 in 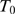 for different levels of 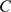. In contrast, 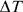 is not linear in 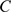 (Fig. 4B), but saturates for high levels of 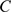.

**Table 1:** Estimates of killing rates based on a model with a saturating killing term for *in vivo* CTL killing data.

	Epitope	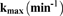	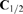	p-value	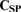	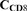
**Effector**	**NP396**	0.0574 (0.049,0.065)	0.0 (0.0,0.005)	0.0041	0.062 (0.054,0.070)	0.119 (0.106,0.132)
	**GP276**	0.0244 (0.008,0.041)	0.0132 (0.0,0.034)	0.0761	0.021 (0.019,0.024)	0.041 (0.037,0.045)
**Memory**	**NP396**	0.0133 (0.0,0.027)	0.0023 (0.0,0.011)	0.0023	0.005 (0.004,0.007)	0.032 (0.024,0.041)
	**GP276**	0.0079 (0.0,0.059)	0.0030 (0.0,0.044)	0.0754	0.004 (0.002,0.005)	0.020 (0.016,0.025)

The parameters 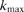 and 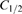 are estimated based on the *in vivo* CTL killing data from Barber et al. [5]. The numbers in brackets represent 95%-confidence intervals based on 1000 bootstrap samples. The 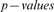 correspond to an 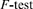 which compares these estimates with the results of fitting the data to a model with a mass-action assumption in the killing term. 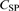 and 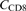 correspond to the mean frequency of epitope-specific CTL measured in the spleen and among CD8^+^ T cells in the spleen, respectively together with their 95%-confidence intervals.

As an improvement over the mass-action killing term, we therefore propose the following relationship between 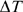 and 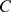:
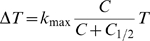
(2)Hereby, 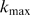 denotes the maximum killing rate at high levels of 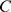, and 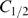 denotes the CTL frequency at which the killing rate is at half of the maximum. Such terms have been suggested previously [25]–[27]. The saturation in the CTL frequency was observed independent of the density of the reticular network (varying the volume occupied by reticular network from 0–50% of the simulated space, *data not shown*). Fitting Eq. (2) to the data generated in our simulations yield 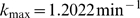 and 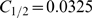. The estimate of 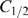 is slightly above the frequency of a subdominant effector or memory CTL response (Tab. 1, see 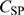 or 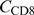, respectively for GP276). This estimate of 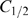 suggests that — if the parametrization of our cellular automaton agrees with the situation *in vivo* — the saturating term Eq. (2) should be preferred over a mass-action killing term for *in vivo* killing data with CTL frequencies 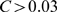.

The result that a killing term which saturates in the CTL frequency is more appropriate stays valid if we include multiple time points at which we calculate the loss in target cell frequency 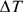. By this, we additionally include the search process and relate to the fact that CTL which are bound in conjugates are prevented from hunting other target cells.

### New estimates of CTL killing rate constants

To account for the non-linear relationship between the loss of target cells and CTL frequencies, we substituted the mass-action killing term in our basic model (see *Materials and Methods*) by one that saturates in 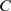. In this case our equation is extended as follows:
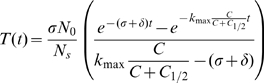
(3)Eq. (3) was fitted to the *in vivo* CTL killing data from Barber et al. [5] as described previously [8].

We obtain a reduction in the residual sum of squares compared to the previous model with a mass-action killing term, Eq. (8). This reduction is significant (as assessed by an 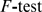) for the effector and memory CTL response against the NP396-LCMV epitope given a significance-level of 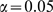 (see Tab. 1). However, even though a saturating killing term does not significantly improve the fit of our killing model to all the data, our simulations strongly suggest that a saturating term is more appropriate to describe the killing dynamics.

We therefore use a saturating term in the following analyses. Estimates for 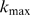 and 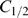 as well as 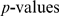 for the 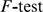 are given in Table 1.

### Influence of single cell behaviour

The saturating term 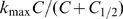 and the term 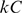 respectively, mathematically describe the reduction in the target cell population due to their interaction with the CTL population. How does this population-level description of the killing dynamics relate to properties at the individual-cell level, such as the velocity of CTL or the time it takes to kill a target cell?

We find that one specific experimental detail is of particular importance for the interpretation of population-level parameters in terms of the single cell properties. This experimental detail concerns the fate of target cells in conjugates after the splenectomy. It is unknown if conjugates are simply broken up by the preparation of the spleen for the cell sorter, or if, during this preparation, killing of target cells that are bound to CTL continues. In any case, conjugates are not observed when the splenocytes are analysed by fluorescent-activated cell sorting (FACS).

### Influence of CTL velocity

We perform simulations with varying CTL velocities and killing durations 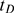. The CTL velocity is indirectly manipulated via the rules of cell movement in our cellular automaton.

**Figure 5:**
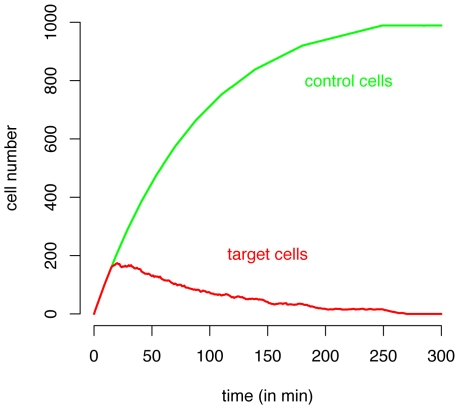
Cell density dynamics for one simulation. The development of the number of target and control cells is shown for a parametrization with 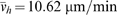 and 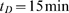.

We generate data with the same structure as Barber et al. [5] (see *Materials and Methods*). We choose a frequency of target-specific CTL with 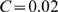 which is orientated at the CTL frequencies observed ([5] and Tab. 1). While the population of target-specific CTL is kept constant according to the assumptions made in the analysis [8],[9], target cells as well as control cells appear in the cellular automaton with a certain rate out of a restricted pool of cells (see Fig. 5 and Video S2 for a time course of one simulation). Each simulation represents the dynamics of CTL killing in a single mouse followed over 300 minutes. We perform 36 simulations for each combination of CTL velocity and killing duration. A bootstrap analysis with 1000 replicates is performed by sampling the number of free and bound target cells, control cells and CTL in 6 randomly chosen “mice” per indicated time-point (at either 15, 30, 60, 90, 120 or 240 minutes) per replicate.

We consider two measures of CTL velocity: (i) 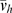, the mean “hunting velocity” based only on CTL that are not bound to target cells, and (ii) 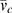, the mean velocity of all CTL regardless of whether they are bound to target cells or not.

**Figure 6:**
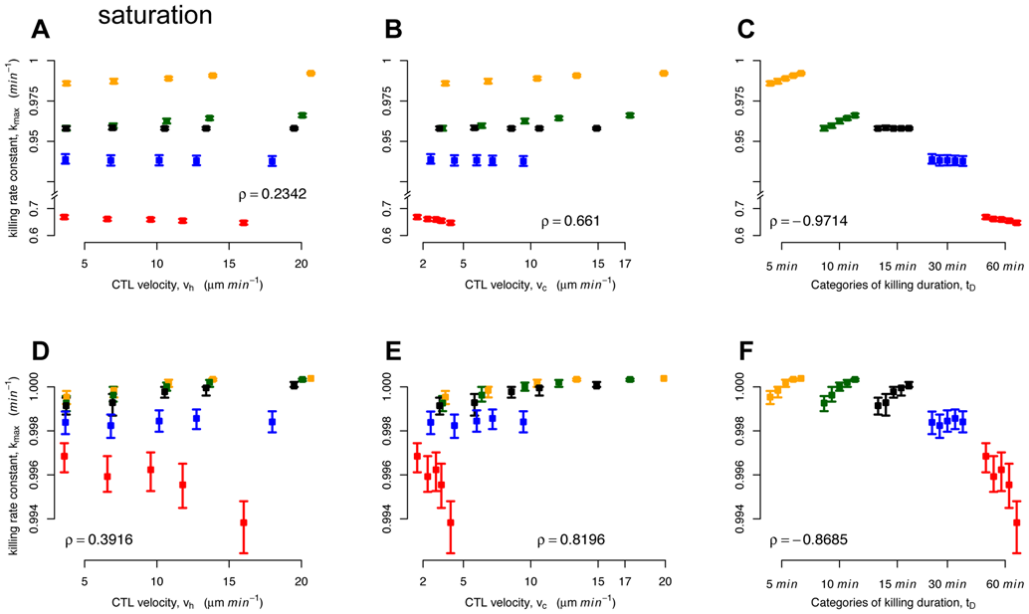
Plots of 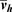, 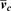 and 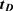 against the estimates of 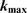. The estimation was based on either counting free and bound target cells (shown in A–C) or only free target cells (shown in D–F). The mean, the minimum and the maximum over 1000 estimates are shown (neglecting outliers) as the 95% confidence intervals are, in most cases, too small to be plotted. Spearman's rank correlation coefficient, 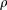, is given. The color coding corresponds to the different levels of 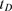 (5 min (orange), 10 min (green), 15 min (black), 30 min (blue), 60 min (red)). In each group of 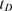 in C and F, the level of velocity is increasing from left to right. Please note that in A–C, the y-axis is split and scaling differs for graphical clarity.

In Fig. 6A, we plot the killing parameter 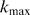 versus the hunting velocity 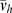. Hereby, we assume that target cells which are bound to CTL remain alive and are counted by the cell sorter. Estimates of Spearman's rank correlation coefficient (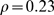) show a weak correlation between 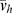 and 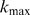 (see Tab. 2). For the mean velocity 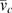 and 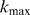, we observe a correlation coefficient of 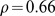 (see Fig. 6B). The finding of a weak influence of the CTL velocity on 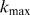 is further corroborated if we analyze the lifespan, 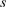, of a single target after it appears in the cellular automaton. For a killing duration of 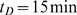, the average lifespan 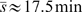, and 80% of all target cells are killed 20 min after their appearance regardless of the CTL velocity. Similar observations are made for the other levels of 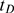. Most target cells are recognized immediately after their appearance in the cellular automaton.

**Table 2:** Correlation between killing rate constants and properties of simulated CTL.

			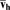	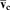	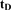
**(a)**	**original**	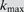	0.2342	0.6610	−0.9714
		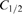	−0.2947	−0.6986	0.9837
		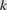	0.2571	0.6814	−0.9798
	**revised**	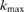	0.2267	0.6664	−0.9784
		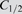	−0.2639	−0.6834	0.9823
		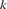	0.4060	0.5888	−0.9696
**(b)**	**original**	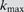	0.3916	0.8196	−0.8685
		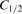	−0.3916	−0.8197	0.8685
		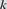	0.4533	0.8379	−0.8753
	**revised**	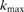	0.6054	0.8711	−0.7895
		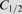	−0.6135	−0.8682	0.7860
		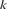	0.5059	0.8628	−0.8688

Spearman's 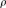 rank correlation coefficient for the estimated killing parameter 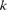 or 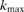 and 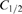 against the CTL hunting velocity 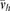, the average CTL velocity 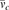 and the killing duration 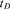, respectively. The estimation was either based on the assumption that targets cells bound in conjugates are counted in the FACS analysis (a) or that they have been killed and therefore not counted (b). Estimates were obtained by using either Eq. (8) (original) or Eq. (9) (revised). For each situation, 1000 data pairs are correlated. (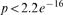 for all coefficients).

Assuming that all target cells bound to CTL are killed before the FACS analysis leads to slightly different conclusions regarding the influence of CTL velocity on the parameter estimates that describe the killing dynamics. We find a stronger correlation between 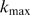 and 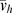 (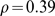), and 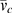 (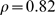) (see Fig. 6D–E).

The correlation between the CTL velocities and 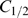 are analogous but inverse to those observed in 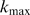.

#### Influence of killing duration 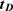


The second property of a CTL, which might influence the estimate of our killing parameters 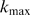 and 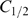, is the time a single CTL needs to kill a target cell, 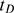. This time includes the establishing of a contact between the CTL and the target cell, the actual killing process and the time required for the detachment of the CTL. Although the conjugate formation and lysis of the target cell can happen very rapidly within 10–25 min, CTL might remain in contact to dead target cells for even longer periods [24],[28],[29]. We choose five different values of 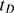 in the range of 5–60 min.

In Fig. 6C, we plot estimates of 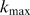 and 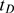, again based on the assumption that free and bound target cells are counted in the FACS analysis. This correlation is the strongest of all the variables: 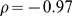. If we assume that only free target cells are counted, we find a slightly lower, but still high, correlation coefficient of 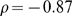 (Fig. 6F). During a simulated time period of 300 min, a CTL kills on average 2–7 target cells depending on the velocity and the killing duration. Thereby, the occurence of multiple killing, where one CTL kills several target cells simultaneously, is rather rare (see Fig. S2). Therefore, we think that this mechanism does not affect our conclusions.

For all the analyses above, we simulate data that reflects the dataset of Barber et al. [5] in terms of number of mice and time-points sampled. To generate such data, we produce detailed time-series of target cells and control cells in each simulation, and then sample only at one time-point. If we use the entire time-series we obtain from a single simulation to estimate 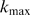 and 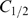, we eliminate the variation in estimates across different simulation runs. With this approach, our conclusions are qualitatively equivalent to those above.

**Figure 7:**
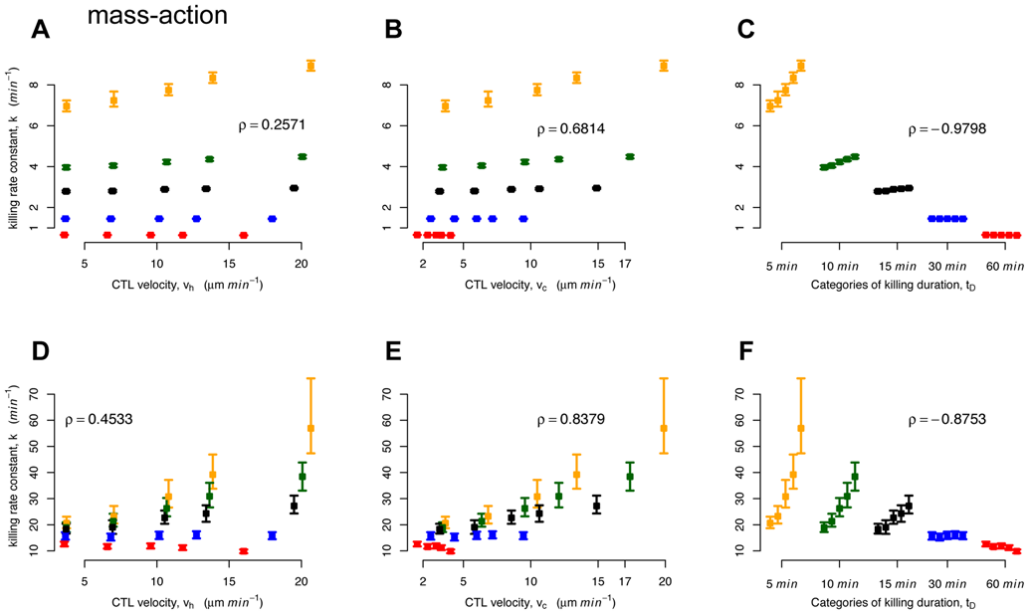
Plots of 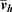, 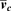 and 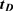 against the estimates of 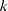. The estimation was based on either counting free and bound target cells (shown in A–C) or only free target cells (shown in D–F). The killing rate constant was estimated using the original method.The mean, the minimum and the maximum over 1000 estimates are shown (neglecting outliers) as the 95% confidence intervals are, in most cases, too small to be plotted. Spearman's rank correlation coefficient, 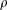, is given. The color coding corresponds to the different levels of 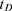 (5 min (orange), 10 min (green), 15 min (black), 30 min (blue), 60 min (red)). In each group of 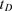 in C and F, the level of velocity is increasing from left to right.

The effect of varying CTL velocities and killing durations on 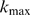 is rather weak in terms of absolute values. The difference which is observed between the estimates of 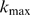 based on simulated and experimental data (Fig. 6 and Tab. 1) might be explained by the varying CTL levels between mice in the experiment which affects the estimation of 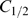 and thus 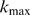. We performed the same analysis with a mass-action term in the killing using either Eq. (8) or Eq. (9) to estimate the per-capita killing rate 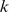. The estimates of 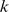 are in the same order of magnitude as in the experiment if we assume that the killing in conjugates does not continue after splenectomy. Given realistic CTL velocities and killing durations 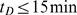, we obtain estimates for 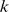 which are close to those estimated based on experimental data [8],[9]. The pattern for 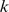 against 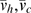 and 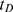 stays the same as for 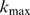 (see Fig. 7 and Fig. S3). The influence of the velocity on the estimates increases given very low killing durations but depends again on the way the target cells are counted. However, the difference in terms of absolute values is more apparent in 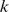 than in 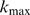.

These general results are not affected by several changes in our assumptions to simulate CTL-target cell interaction. We assumed that a CTL will always recognize a neighbouring target cell and that both cells will immediately stop movement after forming a conjugate. The main influence of the killing duration 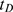 on the killing rate estimates is still observed if we decrease the probability of a CTL to recognize a target cell to 0.5 or allow conjugates to continue migration for a while as observed experimentally [24] (*data not shown*). This observation is also robust to changes in the density of the reticular network varying the density up to 50% of the simulated volume.

## Discussion

Quantifying CTL efficacy based on data of an *in vivo* CTL killing assay requires models which capture the anatomical complexities inside the mice and reflect experimental conditions. Previously, we proposed a population-level model [8],[9], in which the killing dynamics was seperated into migration to and killing in the spleen. However, uncertainties remained concerning the most appropriate mathematical description of the killing term and how to interpret the population-level killing parameter. To address these questions, we constructed a 3-dimensional cellular automaton model of a T cell zone in the spleen.

Unlike previous studies [12],[30], we do not model the biophysical processes involved in cell movement. Rather, we impose simple rules of directed movement and position swapping. We find that our cellular automaton model can recapitulate experimentally observed CTL motility. This has recently been established in a more general context by Bogle and Dunbar [31].

We find that the most appropriate description of the killing term is linear in the frequency of target cells and saturates in the frequency of CTL. This saturation is observed irrespective of the various densities of the reticular network we considered (0%–50%). This is both expected and surprising: Expected, because it is well established that killing terms will saturate in CTL frequency [25],[32] as CTL, while bound to a target cell, are prevented from hunting and killing others. Surprising, because our cellular automaton is spatially structured, and we expected this spatial structure, which was not considered in previous work [25],[32], to percolate into the most appropriate killing term. Additionally, the conditions, under which the saturating term is theoretically derived, namely that there conjugates are in a quasi steady state, are not fulfilled in our simulations.

The main aim of our study was to identify the most appropriate killing term, rather than to obtain new estimates for killing. Nevertheless, we re-analyzed the data by Barber et al. [5] using a model with a saturating killing term. Using a saturating killing term improves the fit significantly for the immunodominant NP396-epitopes in the effector and memory response. This suggests that the CTL levels specific for NP396 are in the saturating regime. The improvement of the fit in the case of the effector NP396 response is consistent with the critical CTL frequency 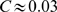 which we derived from our simulations. (Above this critical CTL frequency the killing rate is saturated.) The improvement of the fit in the case of the memory NP396 response is also consistent with the critical CTL frequency if we factor in the finding that memory CTL are mostly located in and around the T cell zones [7]. That means that, in the case of memory responses, it is more appropriate to compare the critical CTL frequency with the proportion of epitope-specific CTL in the pool of CD8^+^ T cells (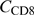 in Tab. 1), rather than the entire spleen (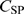 in Tab. 1).

The killing term allows us to estimate populational-level parameters which quantify CTL efficacy. By varying the velocity of CTL and the time a CTL needs to kill a target cell, we were able to determine the influence of these single-cell properties on the population-level killing parameters. We based our analysis on a CTL frequency of 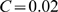 which is in the range of the frequencies observed experimentally ([5] and Tab. 1). We find that the population-level parameters are mostly affected by the killing duration 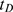. The longer 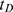, the lower the killing rate constant 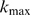 or 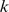. The impact of the CTL velocity on the killing rate constant 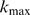 or 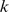 varies depending on our assumptions regarding the fate of target cells in conjugates after the splenectomy. The impact of CTL velocity is weak if we assume that target cells in conjugates are still alive and counted by the cell sorter. If we assume that killing in conjugates continues and, therefore, target cells bound in conjugates are not detected by the cell sorter, the impact of CTL velocity is stronger. To clearly separate the relative effects of killing duration and CTL velocity it is necessary to determine the fate of target cells in conjugates during the *in vivo* killing assay.

Yates et al. [9] showed a significant difference between estimates of the killing rate constant 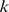 in effector and memory responses. Effector CTL are more efficacious than memory CTL indicated by a higher value of 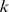. Our analysis suggests that the difference between effector and memory CTL can be explained by a difference in the time 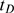 which a single CTL needs to kill a target cell. This hypothesis is in line with the observation that memory CTL store intermediate or low level of perforin and granzyme in comparison to effector CTL, which could prolong the killing process [33]. The difference between the killing rate constants for NP396- and GP276-specific CTL could be explained by different binding rates between the T cell receptor of the CTL and the peptide-MHC complex of the target cells. It is known that the binding of the T cell receptor specific for NP396 to NP396-MHC is stronger than that of the T cell receptor specific for GP276 to GP276-MHC [34]. We find that lower probabilities of recognition, which correspond to low binding rates, lead to lower estimates of 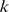 in our simulations (see Fig. S4). To test these hypotheses about the killing process experimentally, one could combine *in vivo* CTL killing assays with two-photon microscopy as it is performed for the analysis of T cell activation [12]–[15],[22],[24].

In a recent study, Ganusov and De Boer [35] calculated target cell half-lives using a mathematical model that did not control for differences in CTL levels. The reason that these authors neglected CTL levels was that the exact form of the killing term is unknown. However, to predict the protection afforded by CD8^+^ T cell responses it is necessary to extrapolate the efficacy of a CTL population of varying size. Further, to decide if effector CTL are more efficacious than memory CTL, or CTL in acute infections are more efficacious than CTL in persistent infections, it is necessary to disentangle quantitative from qualitative aspects. Therefore, the dependence of the killing rate on the level of CTL can, in the long run, not be ignored.

The point of the present study was to derive a more appropriate mathematical description of the killing term from a model that incorporates more of the spatial complexities of the spleen as previous population level descriptions. Based on our analysis, we showed that a killing term which saturates in the CTL frequency would be more appropriate to describe the experimental situation.

There is no clear answer to the question which of the different killing rates for LCMV epitopes presented in this study and published so far [8],[9] should be preferred. Much more detail about the killing process is required to clearly favour one estimate. Our study is a first step to improve the estimation of per-capita killing rates based on a population-level and to enhance their interpretation in terms of single cell properties.

## Materials and Methods

### The cellular automaton

We use a three-dimensional lattice of nodes and edges to simulate the T cell zone of the spleen. Recent analysis showed the suitability of a lattice based approach to simulate T cell movement [31],[36]. We define periodic boundary conditions in which a cell leaving the simulated space on the one side of the lattice will reappear at the opposite side. Each node of the lattice represents a cell or a part of a cell. We consider target cells, target-specific CTL and splenocytes, which occupy a single node. Macrophages and dendritic cells are larger than CTL and have an average diameter of 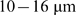 [12],[37]. These cells are modelled as occupying four nodes connected in no regular shape. The shapes are not stable, we only require that each cell-part has at least one other part of the cell as its direct neighbour. Some nodes are occupied by reticular network which does not change position over time and represents spatial obstacles to moving cells. Lastly, a few nodes are left unoccupied and define free space.

Each node has 26 neighbours. As cell movement requires a complex restructuring of the actin cytoskeleton [23], each cell in our cellular automaton is assumed to have a preferred moving direction 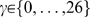. The direction can change upon encounter of another CTL, a target cell, or reticular network (see below). A cell will only have a moving direction of 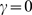 while it is bound in a conjugate. The cellular automaton was implemented in the C++ programming language.

### Cellular automaton - scaling and initialization

We consider a lattice of 30×30×30 nodes, which makes 27000 nodes in total. To set the spatial scale of the simulation we assume that each edge of the lattice has a size of 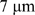, the average diameter of a T cell [38],[39]. The reticular network occupies ≈4500 nodes (≈17% of the space). To achieve the actual structure of a network, we seed the lattice at random nodes from which the network grows until the assigned volume is occupied. As the spleen is a densely packed organ, free space is set to ≈1300 nodes (≈5%). We consider only a small number of large cells (macrophages and dendritic cells) (≈24–40 nodes, <0.1% of space). To determine the appropriateness of the mass-action term, target cells and target-specific CTL are randomly positioned in the lattice according to their assigned frequency. The rest of the lattice is filled with unspecified splenocytes.

For the analysis of the influence of the CTL velocity and killing duration on our estimates, we simulate the migration of target cells into the spleen, in addition to target cell killing in the spleen, in accordance with the events in an *in vivo* CTL killing assay [5]. In these simulations, 450 CTL (≈2% of the total number of cells without reticular network) are randomly positioned in the lattice. Target and control cells (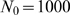 each) appear in the lattice at a rate 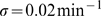 [8]. They can either appear on a free node or replace an unspecified splenocyte, which, in turn, is simply deleted.

At each time-step of the simulation the position and other properties of each cell are updated. A time-step corresponds to 30 seconds of real time. In the simulations in which we increase or decrease the velocity of CTL, we update them more or less often than the other cells, respectively. In these simulations, a time-step corresponds to 12–40 s real time. We initialize our simulations by a burn-in phase of 20 minutes real time before target cells are allowed to migrate into the spleen, and target cells and CTL are allowed to interact. After the burn-in phase a simulation is run for 300 min real time.

The 27000 nodes of the cellular automaton comprise approximately 21000 (biological) cells. As the total number of splenocytes of a mouse spleen is estimated to be around 2×10^7^–10^8^ cells [5],[40], the modelled compartment comprises roughly 0.01%–0.1% of the *white pulp* of the spleen.

### Cell movement

Each cell is able to move. We distinguish between two types of movement. The first is movement into free space. A cell can move into a neighbouring unoccupied node if it has the appropriate moving direction 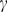. If several cells are able to move into the free spot, one cell is chosen at random. The second type of movement is defined as neighbour swapping. As we are not interested in knowing if an unspecified splenocyte changes its place with another unspecified splenocyte (and to speed up computation), neighbour swapping is performed by target cells and target-specific CTL only. Hereby, such a cell will swap its place with a splenocyte irrespectively of the moving direction 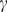 of the splenocyte while two CTL or target cells only swap their places if they move towards each other.

Movement of cells consisting of several nodes involves the restructuring of their shape. Such a cell is simulated to “diffuse” into an unoccupied node by placing its cell-part ( = node) farthest from the unoccupied node into this node. The node which was occupied by the moved cell-part becomes free space. The same procedure is performed for neighbour-swapping with a CTL or target cell. Position changes become effective after all cells updated their position.

If a CTL or target cell is not able to move, it randomly chooses a new moving direction 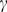. This new direction becomes effective in the next round of updating. The new moving direction is sampled from the set of the 26 possible directions defined by the next neighbours according to the following method. The former moving direction 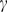 is translated into cartesian coordinates 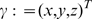, with 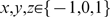. One coordinate 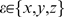 is chosen at random and updated dependent on the former value. As cells prefer small changes in their direction, 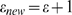 with probability 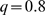 and 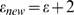 otherwise if 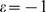 (analogous for 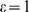). If 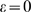, 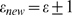 at random. If the cells hit the reticular network, there is a higher chance to make larger turns in our simulations, as there is a high chance that a node in a direction similar to the previous moving direction will also be occupied by reticular network (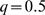). By controling the number of changes and moves per time step, we are able to regulate the velocity of cells. With these rules, CTL will perform random walks as described above (see Fig. 2B).

### CTL scanning and target killing

Before each update of the lattice, all the target-specific CTL scan their direct neighbourhood for target cells. If a CTL encounters a target cell it recognizes it with a certain probability (the probability of recognition). Upon recognition both cells will form a conjugate. Unless it is stated otherwise, we assume the probability of recognition to be one. It is observed, that conjugate-formation is followed by a period where T cells and bound target cells migrate together before they finally stop [24],[41]. However, it is not clear how the direction of the conjugate is determined and what happens if several CTL are bound to one target or vice versa. We assume that conjugates will immediately stop migrating after conjugate formation and stay immobile during the time of the killing, 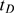. Allowing conjugates to migrate together for a certain time does not generally affect our results.

We allow *multiple killing* of CTL which is in agreement with observations *in vitro* where CTL kill multiple targets simultaneously [28]. When the target cell is killed, the CTL chooses a new moving direction 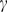 at random and proceeds.

### Quantification of CTL movement and motility

The average velocity of a CTL in a simulation with 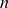 CTL is defined by 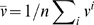, with 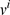 as the average velocity of CTL 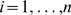 over time. We distinguish between two different types of velocities in the presence of killing. The “hunting” velocity 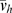 is calculated based on all CTL that are not bound to target cells. The second velocity, 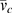, describes the average velocity over all CTL regardless of them being bound to target cells or not.

The motility coefficient 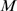 measures the temporal displacement of a cell. If 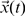 denotes the position of a cell at time 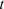 and 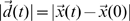 its displacement during this time, the motility coefficient, 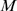, in three dimensions is estimated according to: 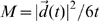. For a graphical representation, we plot the mean displacement against the square root of 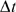, which denotes the time interval on which the calculation of the displacement is based. The motility coefficient can then be calculated from the slope of the curve (Fig. 2C) (see e.g. [18]).

### The *in vivo* CTL killing assay

Our research was motivated by the *in vivo* CTL killing assay presented in Barber et al. [5]. The experimental details are comprehensively described in this paper. Briefly, mice are infected by LCMV to generate CTL responses. Eight days after infection the mice harbour effector CTL, whereas 30 days after infection or later the mice harbour memory CTL. A mixture of fluorescently labelled cells is then injected intravenously into the tail vene of the mice. This mixture consists of equal proportions of target cells expressing either of the two LCMV epitopes (NP396 and GP276) and control cells, which do not express LCMV peptides and are therefore assumed to be unaffected by the CTL response. The frequencies of CTL, target and control cells are measured in the spleen after sacrificing the mice at different time points up to 270 min after the transfer of the target cells.

### The basic model - the dynamic of target cells in blood and spleen

According to previously published mathematical models [8],[9], the data obtained by an *in vivo* CTL killing assay are analysed in two steps. First, we consider the migration of target cells into the spleen after injection. Second, we analyse the killing of target cells in the spleen. The model assumes that killing only occurs in the spleen and that the frequency of target-specific CTL, 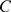, is constant during the short time period of the experiment.

Estimates of migration parameters are based on absolute numbers of control cells in the blood, 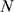, and in the spleen, 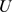. The dynamics is described by:
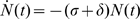
(4)

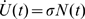
(5)This leads to
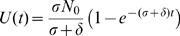
(6)where 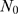 refers to the number of control cells transferred at the start of the experiment. The parameter 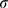 defines the migration rate of cells into the spleen and 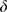 the natural loss rate of cells in the blood. To estimate the actual killing rate 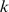, we assume that target and control cells migrate into the spleen following the same rate 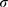.

If 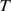 denotes the frequency of target cells in the spleen then the basic model is given by:
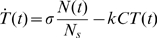
(7)The solution of the above differential equation is:
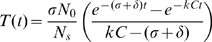
(8)Here 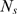 represents the total number of splenocytes.

To fit experimental data to the model, Regoes et al. [8] used Eq. (6) and Eq. (8). Assuming that most of the experimental error arises from different number of cells injected into the mice, this method can be refined [9]. In Yates et al. [9] we used the proportion of target cells that have been killed, 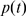, to estimate the killing rate constant 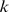. The proportion of target cells that have been killed, 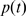, is given by:
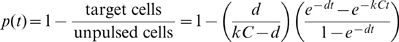
(9)Here 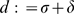.

We showed in Yates et al. [9] that Eq. (9) provides a less biased estimator based on simulated data if there are large variations in the number of injected cells, 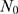. As we control 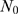 in our simulations, both methods lead to the same results for 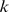 given a mass-action assumption in the killing term (see Fig. 7 and Fig. S3). However, the latter method seems to be less robust if we assume a killing term, which is linear in the target cell frequency and saturates in the CTL frequency. This is surprising as we expect to reduce variation in the estimates with the revised method [9]. We do not understand the lower robustness of the fitting method yet. However, we mainly show results for 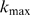 using Eq. (8) because the estimates are more robust for our simulated data.

To perform the statistical analysis we used the 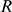 language of statistical computing [42].

## Supporting Information

Figure S1 Distribution for the turning angle θ. The distribution is shown in the absence of killing for the CTL velocities *v_h_* = 3.65 µm/min (A) and *v_h_* = 10.8 µm/min (B) and in the presence of killing for *v_h_* = 10.8 µm/min and *t_D_* = 15 min (C). Reduced velocity as well as killing activity slightly increases the mean turning angle.
(0.10 MB TIF)

Figure S2 Investigating the influence of multiple killing on the simulations. (A) Histogram for the number of target cells killed per CTL for different CTL velocities. Killing duration of *t_D_* = 15 min is fixed and a CTL frequency of C∼0.02 ( = 450 cells) is used. Each simulation comprises 300 min (*v_h_* = 3.73 µm/min (red), *v_h_* = 6.91 µm/min (blue), *v_h_* = 10.54 µm/min (black), *v_h_* = 13.45 µm/min (green), *v_h_* = 19.45 µm/min (orange)). The average number of target cells killed per CTL increases with velocity. (B) The same data as in (A) analyzed for the time a CTL spents to perform multiple killing relative to the time it is bound in a conjugate.
(0.16 MB TIF)

Figure S3 Plots of *v_h_*, *v_c_* and *t_D_* against the estimates of *k*. The estimation was based on either counting free and bound target cells (shown in A–C) or only free target cells (shown in D–F). The killing rate constant was estimated using the revised estimation method based on the proportion of target cells killed. The mean, the minimum and the maximum over 1000 bootstrap estimates are shown (neglecting outliers), as the 95% confidence intervals are, in most cases, too small to be plotted. Spearman's rank correlation coefficient, ρ, is given. The color coding corresponds to the different levels of *t_D_* (5 min (orange), 10 min (green), 15 min (black), 30 min (blue), 60 min (red)). In each group of *t_D_* in C and F, the level of velocity is increasing from left to right.
(0.16 MB TIF)

Figure S4 Estimates for *k* given different probabilities of recognition of CTL for targets. The killing duration and the CTL velocity were kept constant (*v_h_*∼10.8 µm/min, *t_D_* = 15 min). Each dot represents the estimate for one simulation follwed over a time period of 300 min using the revised estimation method based on the proportion of target cells killed. Blue squares represent the mean values per probability of recognition.
(0.11 MB TIF)

Video S1 Movement of simulated CTL in the absence of killing. A time period of 60 min is shown. The CTL velocity is set to *v_h_* = 10.8 µm/min.
(6.90 MB MPG)

Video S2 Dynamics for the interaction of CTL (blue) and target cells (red) for one simulation. Ths simulation was parameterized with *v_h_* = 10.8 µm/min and *t_D_* = 15 min. CTL and target cells bound in conjugates are shown in lightblue and orange, respectively. The movie shows the first 100 min of a simulated time period of 300 min. The size of the full movie is too large to be shown.
(9.43 MB MPG)
