# Supplementary figures and images for: Investigating CTL Mediated Killing with a 3D Cellular Automaton
Source: PLoS Comput Biol. 2009 Aug 21;5(8):e1000466. doi: 10.1371/journal.pcbi.1000466 (PMC2715871; doi:10.1371/journal.pcbi.1000466)

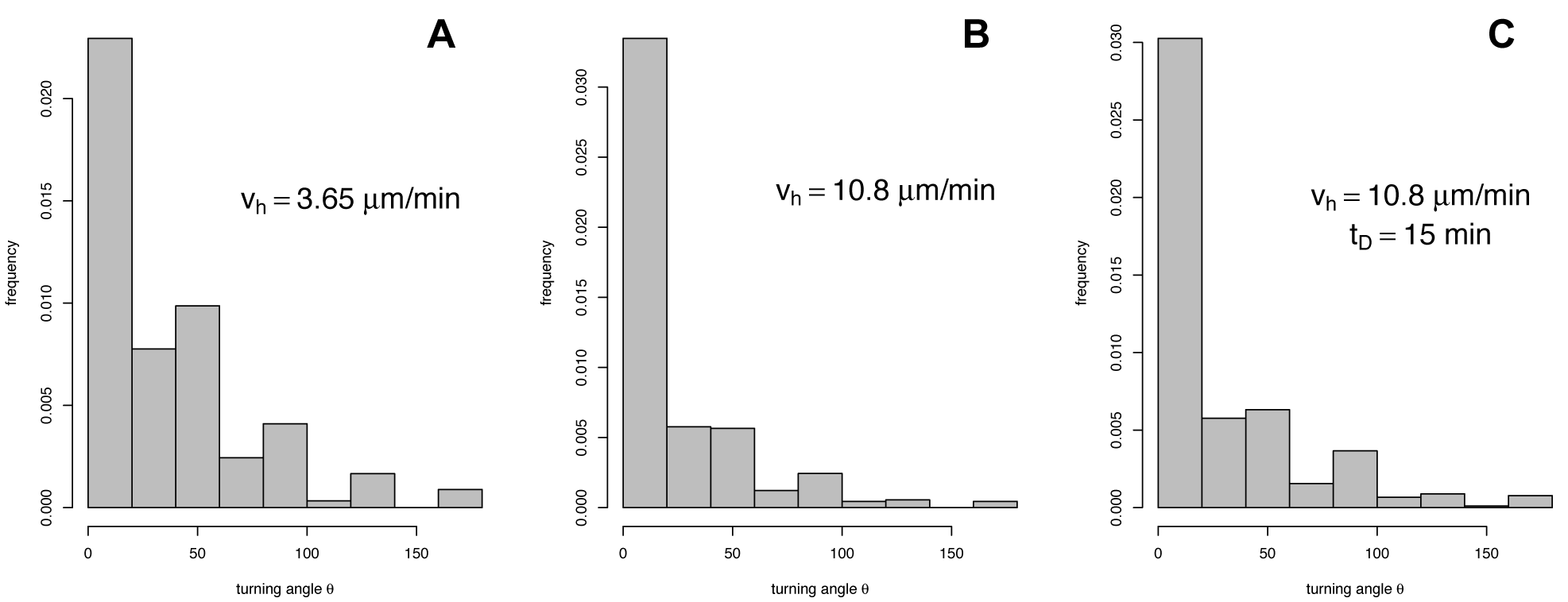

Supplement: Figure S1 — Distribution for the turning angle θ. The distribution is shown in the absence of killing for the CTL velocities vh = 3.65 µm/min (A) and vh = 10.8 µm/min (B) and in the presence of killing for vh = 10.8 µm/min and tD = 15 min (C). Reduced velocity as well as killing activity slightly increases the mean turning angle. (0.10 MB TIF) [file pcbi.1000466.s001.tif]

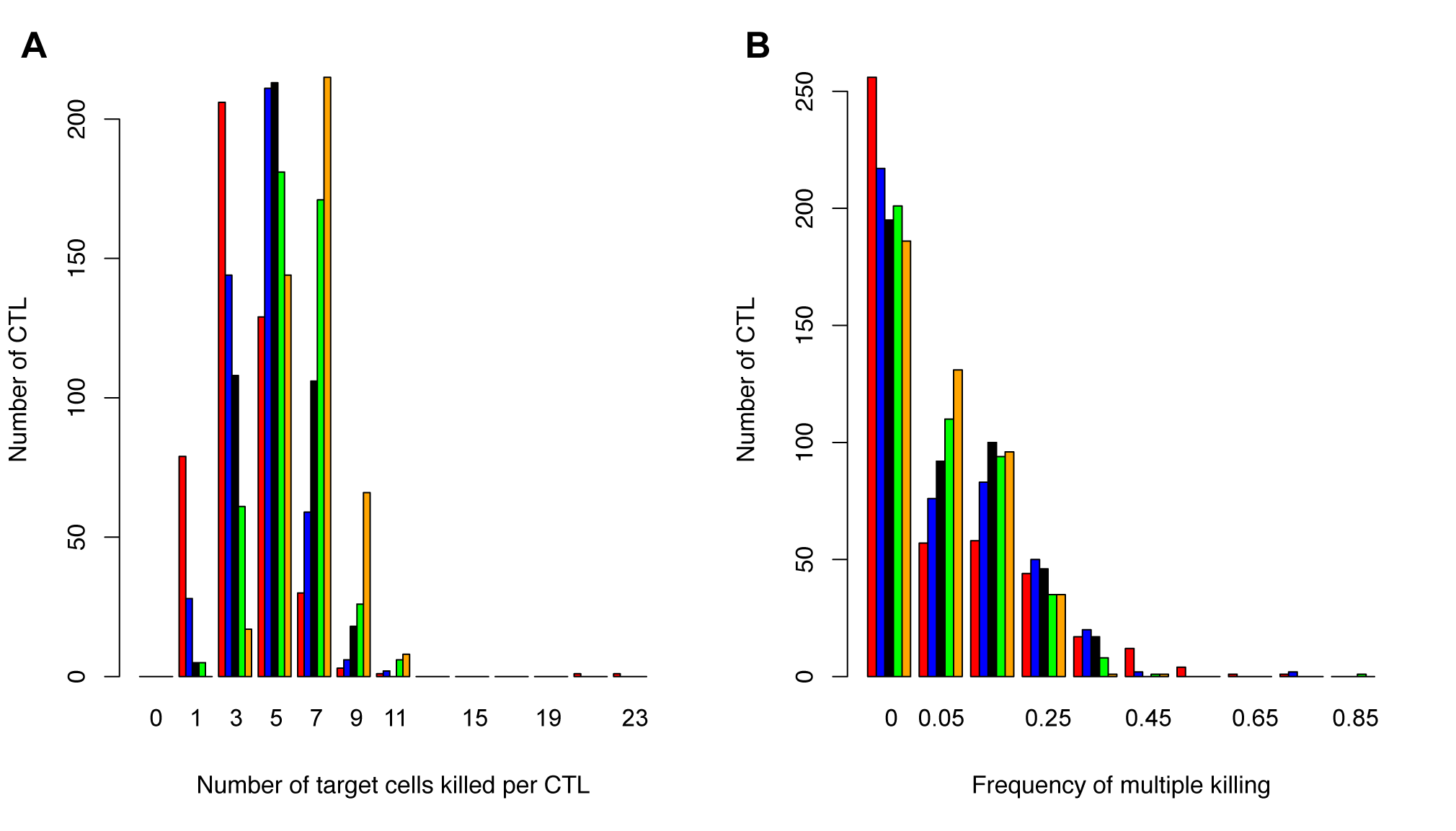

Supplement: Figure S2 — Investigating the influence of multiple killing on the simulations. (A) Histogram for the number of target cells killed per CTL for different CTL velocities. Killing duration of tD = 15 min is fixed and a CTL frequency of C∼0.02 ( = 450 cells) is used. Each simulation comprises 300 min (vh = 3.73 µm/min (red), vh = 6.91 µm/min (blue), vh = 10.54 µm/min (black), vh = 13.45 µm/min (green), vh = 19.45 µm/min (orange)). The average number of target cells killed per CTL increases with velocity. (B) The same data as in (A) analyzed for the time a CTL spents to perform multiple killing relative to the time it is bound in a conjugate. (0.16 MB TIF) [file pcbi.1000466.s002.tif]

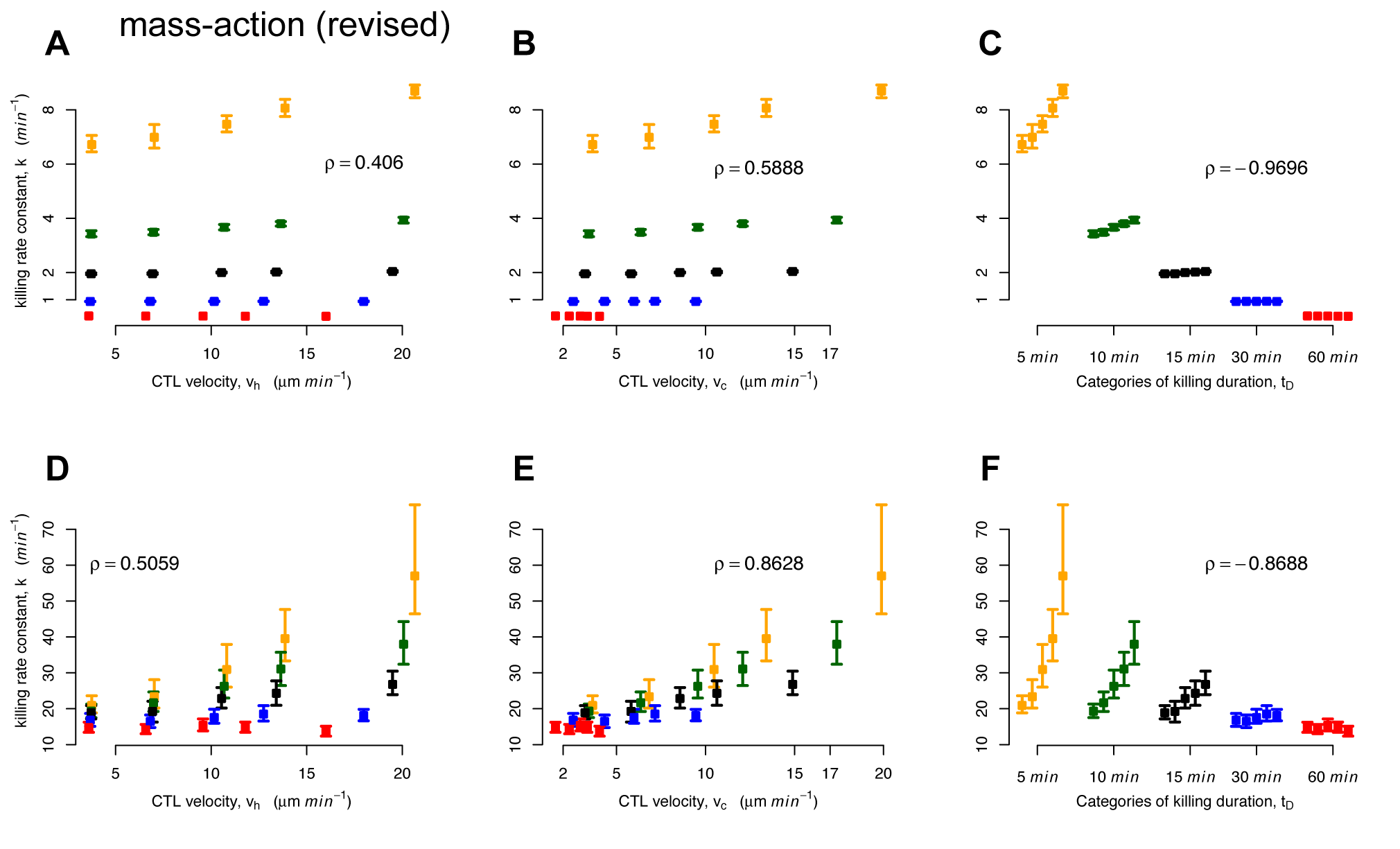

Supplement: Figure S3 — Plots of vh, vc and tD against the estimates of k. The estimation was based on either counting free and bound target cells (shown in A–C) or only free target cells (shown in D–F). The killing rate constant was estimated using the revised estimation method based on the proportion of target cells killed. The mean, the minimum and the maximum over 1000 bootstrap estimates are shown (neglecting outliers), as the 95% confidence intervals are, in most cases, too small to be plotted. Spearman's rank correlation coefficient, ρ, is given. The color coding corresponds to the different levels of tD (5 min (orange), 10 min (green), 15 min (black), 30 min (blue), 60 min (red)). In each group of tD in C and F, the level of velocity is increasing from left to right. (0.16 MB TIF) [file pcbi.1000466.s003.tif]

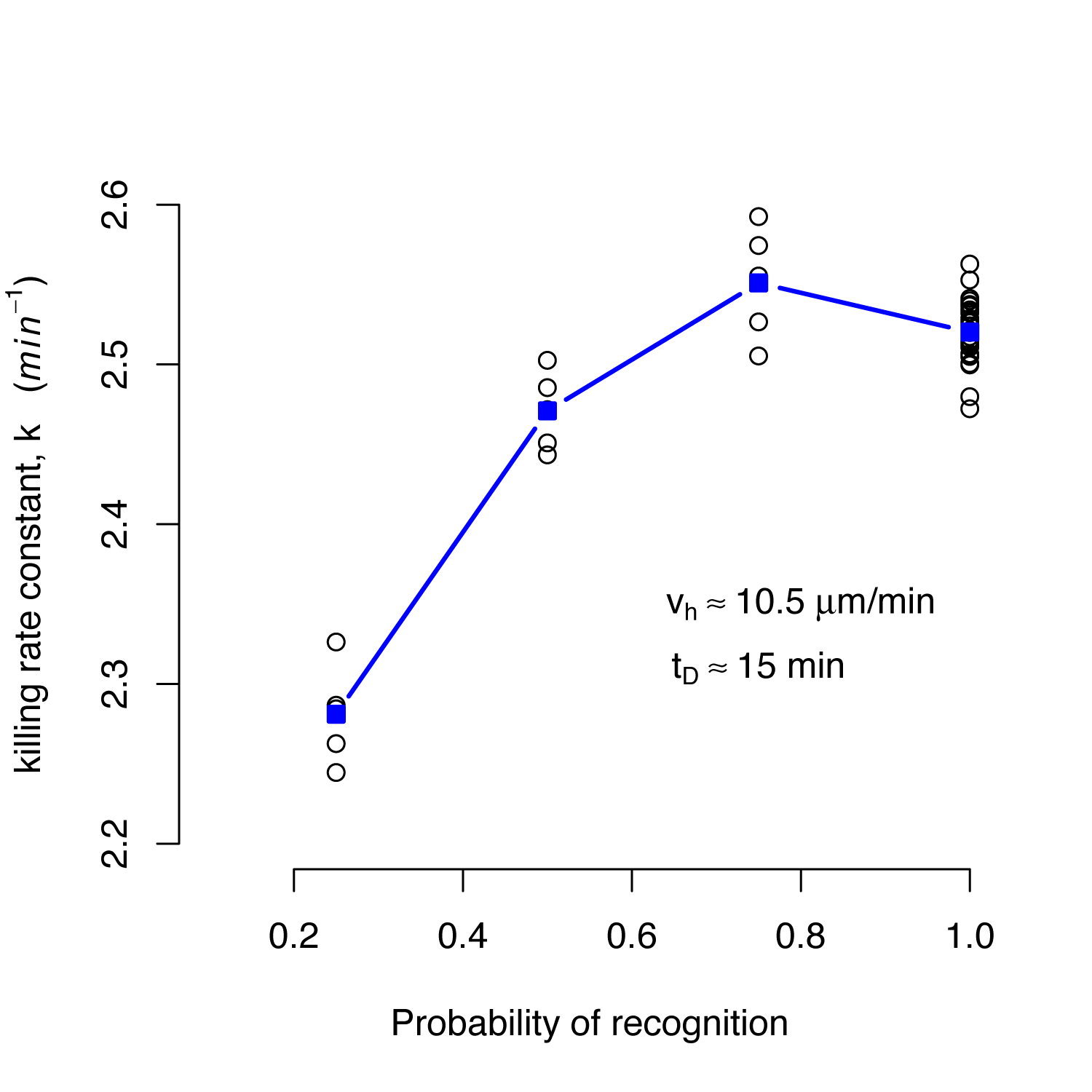

Supplement: Figure S4 — Estimates for k given different probabilities of recognition of CTL for targets. The killing duration and the CTL velocity were kept constant (vh∼10.8 µm/min, tD = 15 min). Each dot represents the estimate for one simulation follwed over a time period of 300 min using the revised estimation method based on the proportion of target cells killed. Blue squares represent the mean values per probability of recognition. (0.11 MB TIF) [file pcbi.1000466.s004.tif]
